# Case of a Schirrous Affection of the Stomach; with an Account of the Appearances on Dissection

**Published:** 1790

**Authors:** William Loftie

**Affiliations:** Surgeon at Canterbury


II. *Caſe of a ſchirrous Affection of the Stomach; with an Account of the Appearances on Diſſection. By Mr.* William Loftie, *Surgeon at Canterbury. Communicated in a Letter to* John Hunter, *Eſq. F. R. S., and by him to Dr.* Simmons.

MRS. G., aged forty years, had been ill for two or three years. Her general complaints were frequent vomitings, pain in the region of the ſtomach and abdomen, and obſtinate coſtiveneſs. From the dark colour of the vomitings, and the black pitchy nature of the ſtools, her caſe was ſuppoſed to ariſe from ſome diſorder of the liver or biliary veſſels, and medicines of every kind, which might tend to remove obſtructions in thoſe parts, were recommended. No relief being experienced from a long trial of theſe, ſhe was ſent to Bath, but did not long remain there, as the waters were ſoon found to diſagree with her. On her return to London, among other things, crude quickſilver was given to her; but not finding herſelf in the leaſt better, ſhe ſoon laid it aſide, and returned home.

A tumour, at this time firſt noticed in the abdomen, was ſuſpected to ariſe from a ſchirrous ſtate of the liver; but, upon a more ſtrict and careful examination, I was convinced that it was not an enlargement of that viſcus. It was ſituated rather on the left ſide of the navel, was moveable and detached, of an oblong, irregular form, and rather hard ſubſtance. By gentle preſſure it might almoſt be brought to the region of the ſtomach; and when the ſickneſs or retching came on, it proceeded from that part. The ſize of the tumour, as it appeared through the muſcles, was about four inches in length, and two or three in thickneſs, and was very evident to the fight on ftripping the body. The menftrual difcharge continued tolerably regular till within a few months of her death. By the long continuance of her illnefs, fhe was more emaciated than I ever faw, or even fuppofed the human body could be, and I may juftly fay fhe was, in the ftricteft fenfe of the word, ftarved.

The following is a ftate of the appearance of the vifcera upon opening the body:

On laying the abdomen open, we did not at firft fight difcover the omentum; but a confiderable tumour appeared rather on the left fide, and very little above the navel, which, on examination, was found to be the ftomach. The mefentery was fchirrous through its whole extent; and the glands of the inteftines were in the fame ftate. The colon firmly adhered to the lower and under part of the ftomach; and the omentum, being much contracted, formed a granulated, and, in fome places, an almoft cartilaginous fubftance ftrongly connected with the ftomach. The ftomach, however, was the feat of the difeafe. The cardia and upper part were found; but the lower part was one uniform fchirrus, extending to within an inch of the pylorus, which was in a natural ftate. The tumour, which in ſome places was an inch and a half in thickneſs, and in other parts half an inch, almoſt prevented even liquids from paſſing into the duodenum. The liver was perfectly ſound. The gall bladder contained a quantity of bile. The ſpleen ſeemed rather ſmaller than natural. The ſmall inteſtines ſhewed ſome appearance of inflammation, though ſhe did not complain of any pain for ſome days before death. The lungs adhered to the pleura, and at places ſhewed the ſame kind of indurations as the meſentery. There was about half a pint of water in the abdomen. The inteſtines, from the long continuance of the diſorder, and the very ſmall quantity of food that had paſſed into them, could not aſſiſt in ſupporting the ſtomach in its proper ſituation; and its increaſed weight, from the ſchirrus, might occaſion its ſinking and forming the uncommon tumour in the abdomen.

One thing neceſſary to be obſerved as rather extraordinary is, that the vomiting ceaſed ſome days before death, though, during that time, ſhe took rather more nouriſhment. A diarrhœa ſupervening might account for the ceſſation of the vomiting, but certainly haſtened her death.

Bonetus

Bonetus * has recorded two or three cafes of fchirrous tumours of the ftomach ; but they do not feem to come up to the prefent cafe either in fubftance or effect.

Morgagni, in his great work *de Sed. et Caufis Morborum*, has likewife collected many inftances of difeafed and mifplaced ftomachs, only one of which feems analogous to the cafe I have been defcribing. In the 65th letter of that work, article 2, he quotes, from Langguthus, a cafe attended with frequent vomiting, from the coats of the ftomach being thickened to an immenfe degree, and become fchirrous, fo as every where to aftringe the pylorus, and render it very narrow ; and in the fame letter, article 15, referring again to the fame cafe, he farther obferves, that the tumour, by its weight, had moved the ftomach from its place, and made the upper orifice thereof be diftant from the diaphragm the fpace of a large fpan ; at the fame time, that the fundus was ftretched out below the navel into the hypogaftric region.

*Canterbury*,
June 18, 1789.

* Vide Bonet. Sepulchret. Tom. II. p. 773.

III. *Farther*

